# A Reliable Fault Diagnosis Method for a Gearbox System with Varying Rotational Speeds

**DOI:** 10.3390/s20113105

**Published:** 2020-05-31

**Authors:** Cong Dai Nguyen, Alexander Prosvirin, Jong-Myon Kim

**Affiliations:** School of Electrical, Electronics and Computer Engineering, University of Ulsan, Ulsan 44610, Korea; daimtavn@gmail.com (C.D.N.); a.prosvirin@hotmail.com (A.P.)

**Keywords:** adaptive noise reducer, gaussian reference signal, gearbox fault diagnosis, one against on multiclass support vector machine, varying rotational speed

## Abstract

The vibration signals of gearbox gear fault signatures are informative components that can be used for gearbox fault diagnosis and early fault detection. However, the vibration signals are normally non-linear and non-stationary, and they contain background noise caused by data acquisition systems and the interference of other machine elements. Especially in conditions with varying rotational speeds, the informative components are blended with complex, unwanted components inside the vibration signal. Thus, to use the informative components from a vibration signal for gearbox fault diagnosis, the noise needs to be properly distilled from the informational signal as much as possible before analysis. This paper proposes a novel gearbox fault diagnosis method based on an adaptive noise reducer–based Gaussian reference signal (ANR-GRS) technique that can significantly reduce noise and improve classification from a one-against-one, multiclass support vector machine (OAOMCSVM) for the fault types of a gearbox. The ANR-GRS processes the shaft rotation speed to access and remove noise components in the narrowbands between two consecutive sideband frequencies along the frequency spectrum of a vibration signal, enabling the removal of enormous noise components with minimal distortion to the informative signal. The optimal output signal from the ANR-GRS is then extracted into many signal feature vectors to generate a qualified classification dataset. Finally, the OAOMCSVM classifies the health states of an experimental gearbox using the dataset of extracted features. The signal processing and classification paths are generated using the experimental testbed. The results indicate that the proposed method is reliable for fault diagnosis in a varying rotational speed gearbox system.

## 1. Introduction

Gearboxes are widely used in industrial applications, usually in harsh and continuous conditions, making them susceptible to a variety of failures. Defects can cause the gearbox system to break down and potentially damage complex mechatronic equipment or even cause a serious threat to safety, property, or customer satisfaction. Therefore, it is essential to diagnose gearbox faults regularly to ensure their early detection. The vibrations of gearbox systems have been studied since the 1980s, and previous researchers have found that gearbox vibrations have a keynote meshing frequency [1,2] with complex sidebands around it and its harmonics [3,4]. Therefore, the sideband frequencies and the meshing frequency and its harmonics are the informative components for identifying gear faults. Signal analysis is a backbone procedure for rotational-machine fault diagnosis research and applications. It works by decomposing the related fault features that are the groundwork for identifying fault patterns. The vibration characteristics of gearbox systems produce two major signals that can be analyzed for fault detection: acoustic signals and vibration signals [5]. Vibration signals are the most popularly used ones for gearbox fault monitoring because acquiring vibration data is easy [6]. However, vibration signals contain many types of noise from sources such as measurement systems (data acquisition systems), the environment, shafts, gears, and other related components and their impingement [7,8]. All that noise, which exceeds the signal, fills the frequency spectrum of the vibration signal, and eclipses it.

Many signal processing methods using advanced techniques have been presented by many researchers: frequency analysis focusing on Fourier transform [9], Wigner distribution [10], rank-order morphological filter [11], cyclostationary signals for mechanical applications [12], and the envelope analysis [13], which is the most well-known for rotational-machine fault diagnosis applications such as bearing-fault diagnosis. It detects the repeating shock amplitudes that appear as faulty teeth traverse each rotation cycle. Using this method, the vibration signal is first processed by a bandpass filter to achieve a high signal-to-noise ratio, and second, the Hilbert transform is used to achieve the envelope. If the sideband frequencies in a gearbox vibration signal appear in the envelope, the presence of faulty teeth in the gearbox can be deduced [14,15]. However, when the vibration signal is submerged in noise, it is difficult to recognize the informative components for fault diagnosis in the envelope. 

Time-frequency analyses were developed to process non-stationary signals using a frequency transformation process divided based on windows across the time axis to capture informative events. The basic time-frequency analysis method is a short-time Fourier transform (STHT) or a spectrogram, such as a limited time window–width Fourier spectral analysis [16,17]. The challenges of the STHT method, such as a failure of the assumption that the pieces of a non-stationary signal are stationary, difficulty adapting the observation window size to the size of a real stationary piece of signal, and the conflict between frequency resolution and time resolution (which is related to the Heisenberg uncertainty principle) limit its usability. To resolve the disadvantages of the STHT method, the wavelet approach was developed as an adjustable window frequency spectral analysis method. The basic wavelet function can be modified to meet special needs, so the wavelet transform produces outputs with good resolution in the low-frequency range and good time resolution in the high-frequency range [18,19,20]. In the region relevant for rotational-machine fault diagnosis, wavelet-based decomposition has been widely used to apprehend the useful components of a vibration signal in a non-stationary condition (in this context, *non-stationary* is the notion that the sideband frequency information of a vibration signal is time-variant). Wavelet transform decomposes a vibration signal into many sub-bands that express the time-frequency distribution through the dilation and transition of the mother wavelet. The sub-bands that contain fault-related intrinsic features can then be used in the fault diagnosis process [21,22]. Nevertheless, the efficiency of the wavelet-based method correlates with the basic wavelet function, so informative components that do not correlate as well with the applied wavelet could be missed or lost in the transformed outcome. In addition, the white noise that is frequently parasitic in a vibration signal and appears across the whole range of the frequency spectrum gives correlated oscillations with a high potential to appear as excitation. In this paper, the effect of noise on the wavelet applied to the wavelet transform method is compared with the proposed method for processing the signal path in the experimental results.

The Hilbert-Huang transform (HHT) was introduced as a better methodology for analyzing non-linear and non-stationary signals [23]. This technique is now often used for rotational-machine fault diagnosis [24,25,26,27]. The HHT method uses a time adaptive operation known as empirical mode decomposition (EMD) to decompose the signal into a group of complete and orthogonal components, denoted as intrinsic mode functions (IMFs), that represent the intrinsic oscillation modes of the fault-related components of a vibration signal. The HHT method was shown to outperform wavelet transform in rotational-machine fault diagnosis in [28,29,30]. To capture the advantages of the HHT, several fault detection tools combine EMD with other methods, such as envelope analysis and the wavelet-based technique. EMD and the envelope analysis combine in series: the vibration signal is first decomposed by EMD to determine the number of IMFs; the envelope analysis then processes the IMFs to monitor for fault-related components. Compared with previous methodologies, this combined technique had better results [28,31,32]. Combining wavelet transform and EMD for time-frequency analysis is another currently used combination method. It takes advantage of the strong points of the two techniques and minimizes their limitations, particularly aliasing in the high-frequency band (wavelet transform) and difficulties in isolating the signals within the second harmonic (EMD) [33,34]. 

However, the EMD technique is sensitive to noise, so noise-related IMFs, which are not useful, can be generated by the EMD. As illustrated by Van M. et al. [35], EMD performs well in processing low-noise vibration signals and poorly in processing high-noise signals. In other words, even EMD combination techniques are unreliable in noisy environments. Therefore, to effectively apply enhanced signal analysis techniques to non-stationary vibration signals, a proper pre-processing method to reduce noise is required, such as narrowband demodulation [36] or discrete wavelet transform (DWT) [37,38]. Applying those de-noising methods effectively reduces the measurement noise, but the original informative signal is also distorted by the attenuation of a narrow bandpass filter in narrowband demodulation or the threshold in DWT-based de-noising. In other words, using one of those noise reduction methods can degrade the performance of a fault diagnosis system. Therefore, we have developed a new de-noising technique to reduce the noise from an original vibration signal by optimizing a process for filtering the weights and parameters of the reference input signals (adaptive) and considering the noise characteristics and rotational speed. We call our new technique the Adaptive Noise Reducer–based Gaussian Reference Signal (ANR-GRS). 

The adaptive noise-controlling technique reduces noise by means of destructive interference. It consists of an adaptive filter and reference signals. The adaptive noise filter is a digital filter with an adaptive algorithm that adjusts the filtering coefficients (or tap weights) so the filter can be flexibly and optimally operated in unknown conditions with non-stationary signals to effectively remove low-level noise [39]. The typical performance criterion for adjusting the filtering weights (convergence condition) is based on the error signal, which is the difference between the output of the filter and the input reference signal as determined using the recursive least-squares or least mean square (LMS) algorithm. Between them, the LMS is more widely used because of its robustness and simplicity [40]. The ANR-GRS technique has three main function blocks: Gaussian reference signal (GRS) generation, adaptive noise filtering using the LMS algorithm, and optimal output sub-band selection. The generated GRS is a special signal consisting of a white-noise reference signal and a Gaussian reference signal with adjustable parameters (mean value and standard deviation) to identify noise components that are independent of the informative components in the frequency domains of the vibration signal from a varying speed gearbox. The adaptive noise filter consists of an M-tap digital Finite impulse response (FIR) filter and the LMS adaptive algorithm; it has two inputs: a reference input for the GRS signal with specific parameters and the desired input for a vibration signal. The noise-reduced sub-band is achieved as the output of the adaptive noise filter. The optimal output sub-band selection adjusts the parameter of the GRS signals to receive the set of noise-filtered sub-bands output by the adaptive filters and then selects the sub-band with the minimum mean square as the optimal sub-band, which is the final output of the ANR-GRS module. That output becomes the input for the feature pool configuration process used to extract the statistical features in the time and frequency domains of the vibration signal as feature vectors [41] to be classified. 

The heterogeneous feature pool improves the efficiency of gearbox fault expression for fault diagnosis process; however, the high dimensionality of the feature vectors can be a challenge for various machine learning techniques that can be used for decision making. In comparison with the other artificial intelligence algorithms, the classification performance of support vector machines (SVM) classifier is not much sensitive to the dimensionality of the feature vectors, in other words, this algorithm is not affected by the problem called ‘curse of dimensionality’. Furthermore, SVM demonstrates excellent generalization performance, so this technique is capable of achieving high accuracy while classifying mechanical faults in rotation machinery [42]. Also, with an appropriate kernel function, SVM can accurately separate the non-linear datasets by hyperplanes in high-dimensional feature space using the non-linear mapping [43]. SVMs are widely used for fault diagnosis in many real-world applications [44]. They were originally designed for binary classification and then improved for multiclass classification using the one against one, one against all, or hierarchical strategy. Among them, the one against one strategy is the most reliable for our purposes [45,46,47]. Therefore, a one-against-one multiclass SVM (OAOMCSVM) is used in this proposed methodology. 

The new hybrid technique employs the ANR-GRS, which produces an optimal sub-band, and then uses a machine-learning classification of fault types based on the OAOMCSVM on features extracted from that optimal subband to identify faults in a gearbox system. The experimental results show that the proposed method outperforms the aforementioned denoising methods, which verifies that the “clean” input can be used to produce correct output from the signal processing and classification paths.

The rest of this paper is organized as follows: Section 2 provides the characteristics of a gearbox vibration signal and the experimental test setup used in this study. The proposed methodology is explained in detail, from theory to the construction of the ANR-GRS, feature pool configuration, and OAOMCSVM classification, in Section 3. Section 4 demonstrates our experimental results in signal processing and classification. Finally, Section 5 concludes the paper.

## 2. The Characteristics of a Gearbox Vibration Signal and Experimental Testbed Setup

### 2.1. The Characteristics of a Gearbox Vibration Signal

**Figure 1:**
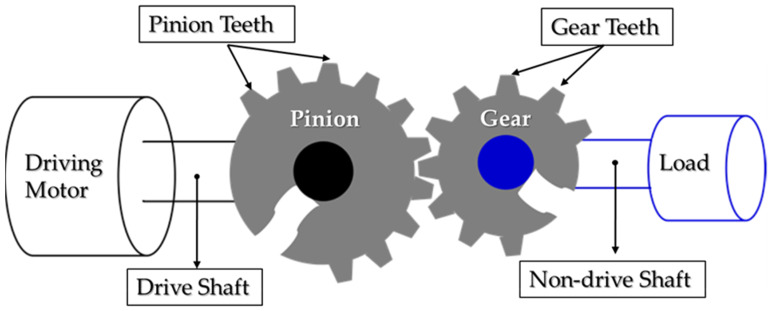
The spur gearbox model.

**Figure 2:**
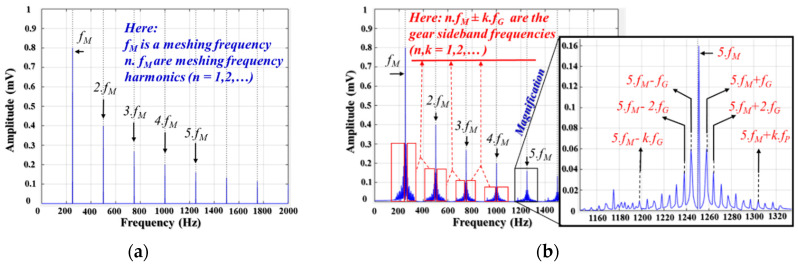
The frequency spectrum of the gearbox vibration signal: (**a**) a healthy gearbox and (**b**) a faulty gearbox.

The defects of a gearbox can be classified into three major categories: manufacturing defects (tooth profile error, eccentricity of the wheel, etc.), installation defects (parallelism), and operational defects (tooth wear, case wear, tooth spalling, tooth cracks). This research considers operational faults. A one-stage transmission gearbox, which consists of two rigid blocks with a pinion (on the drive side) and a gear (on the non-drive side), is illustrated in Figure 1. A healthy gear in normal condition working smoothly and periodically generates a linear and periodic vibration signal [3]. The vibration signal, $s_{h}$[mV/(m/s^2^)], of a fault-free normal pair of gears meshing under a constant load speed can be formulated as [48]:

(1) $$s_{h}\left(t\right)=\overset{N}{\underset{i=0}{\sum }}S_{i}\cos\left(2\pi if_{M}t+\varphi_{i}\right),$$

where $S_{i}$ and $\varphi_{i}$ are the amplitude and phase of the i-*th* meshing frequency harmonics; $f_{M}$ is the meshing frequency (for the pinion: P is the number of teeth in the pinion wheel, $f_{P}$ is the pinion rotation frequency, $f_{M}=P.f_{P}$; or $f_{M}=G.f_{G},\;G$ is the number of teeth in the gear wheel, $f_{G}$ is the gear rotation frequency), and N is the total number of $f_{M}$ harmonics in the frequency range of a vibration signal. Figure 2a shows the spectrum of the output vibration signal of a fault-free gearbox; it is filled with the frequency tones of the meshing frequency and its harmonics.

In a fault case, when the motion transferred from the drive shaft to the non-drive shaft by the rotation between the pinion wheel and the gear wheel traverses a defective tooth (chipped, worn, or missing), an abnormal movement occurs that changes the impulses in the vibration signals. The vibration signal contains amplitude and phase modulations of the carrier frequency as the meshing frequency; its frequency spectrum includes sidebands, frequency components on two sides of the meshing frequency and its harmonics, as given in Equation (1). Thus, when the gear wheel has a faulty tooth, the velocity of the gear angle changes impulsively within the rotating functionality and generates a non-linear vibration signal in which issues such as speed variation, amplitude, and phase modulation prevail [4]. The vibration signal is formulated [3] as given by Equation (2), and an example of its spectrum is shown in Figure 2b:

(2) $$s_{f}\left(t\right)=\overset{N}{\underset{k=0}{\sum }}S_{k}\left(1+a_{k}\left(t\right)\right)\cos\left(2\pi kf_{M}t+\varphi_{k}+p_{k}\left(t\right)\right)$$

Here, $a_{k}\left(t\right)=\sum_{j=0}^{M}A_{kj}\cos\left(2\pi jf_{G}t+{µ}_{kj}\right)$ and $p_{k}\left(t\right)=\sum_{j=0}^{M}P_{kj}\cos\left(2\pi jf_{G}t+\xi_{kj}\right)$.

$A_{kj}$, $P_{kj}$ are amplitudes and ${µ}_{kj}$, $\xi_{kj}$ are phases of the j-*th* sideband in the amplitude and phase modulation signals, respectively, around k meshing harmonics. 

### 2.2. The Experimental Testbed Setup

**Figure 3:**
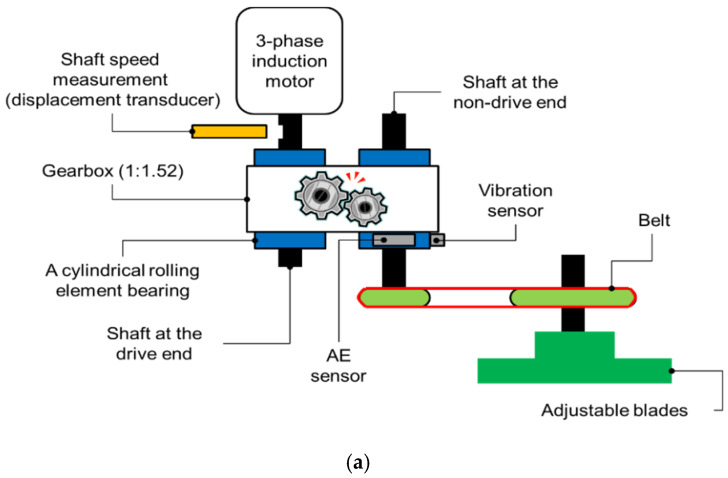

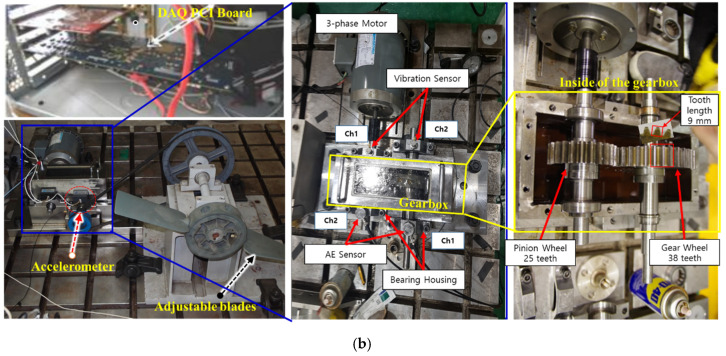
Experimental testbed setup: (**a**) function block diagram; (**b**) actual experimental assembly.

The experimental testbed is illustrated in Figure 3. The pinion wheel is fixed to a three-phase AC induction motor by a drive shaft (DS). The motion (torque) is transmitted from the AC motor to the load as adjustable blades, which are mounted on the end of the non-drive shaft by the engaged teeth of a pinion wheel and a gear wheel (a gearbox with a gear reduction ratio of 1:1.52). 

**Figure 4:**
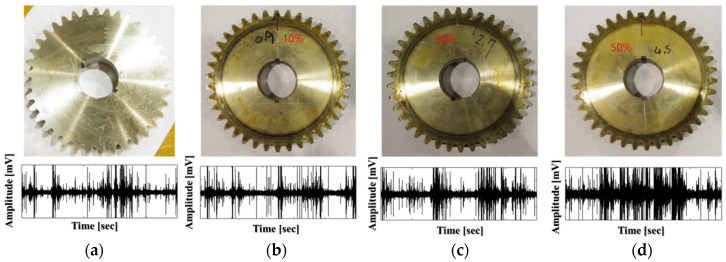
The health states of the gear wheel and examples of vibration signals at the rotation speed of 300 RPM: (**a**) no seeded fault, healthy gear, (**b**) tooth cut 10% (0.9 mm), (**c**) tooth cut 30% (2.7 mm), (**d**) tooth cut 50% (4.5 mm).

The number of teeth on the pinion wheel is 25 (P = 25), the gear wheel has 38 (G = 38), and the length of each tooth is 9 mm. Figure 4 depicts the seeded tooth failures on the gear wheel: a perfect or healthy gear (H), tooth cut 10% (F1), tooth cut 30% (F2), and tooth cut 50% (F3). To measure the speed of the shaft rotation, a displacement transducer is placed to track the hole in the DS once per rotation. The vibration signals from the gear wheel in the normal condition and three levels of tooth cut defects (shown in Figure 4) were continuously acquired from the vibration sensor (accelerometer 622B01 made by the IMI Sensor Company) mounted on the end of the DS, 72.5 mm from the pinion gear. The analog vibration was digitized using PCI-2 data acquisition (the specifications of the data acquisition system are provided in Table 1). The sample datasets for each health condition (H, F1, F2, F3) of the gearbox under four shaft speeds are provided in Table 2. 

Each health state is sampled by sampling frequency 65536 Hz in continuous 1 s (1-s sample) repeating by 300 times to receive 300 1-s samples for each shaft speed. Hence, the number of samples for each health state is 1200 vibration samples in four different shaft speeds, the total number of samples in this experimental testbed is 4800 of 1-s samples.

## 3. The Gearbox Fault Diagnosis Methodology

**Figure 5:**
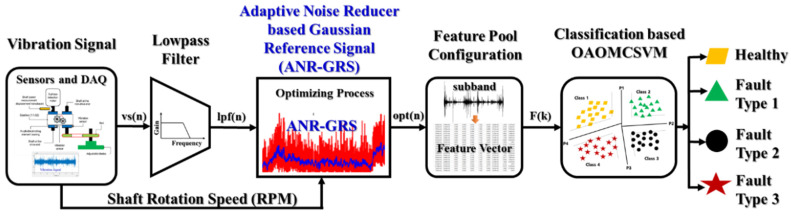
Function block diagram of the proposed methodology.

A function block diagram of the method for gearbox fault diagnosis proposed in this study is provided in Figure 5. We use four main processing blocks: the sensor and DAQ block, ANR-GRS, feature pool configuration, and multiclass SVM-based classification. To acquire discrete samples of each captured signal event containing information about the defective gearbox in the acquisition dataset, the raw vibration signal was sampled at a high frequency of 65536 Hz to acquire rich digitized vibration sample data under different shaft rotation speeds (300, 600, 900 and 1200 RPM), and the adjustable load was non-stationary. Though the operation frequency range of a vibration sensor in this study is from 0.42 Hz to 10 kHz (this is presented in Table 1), thus fault-related components in the frequency domain of vibration signals mostly exist in the lowest segment 0–10 kHz of their frequency spectrums. Therefore, the sampling frequency of the raw vibration signal (all vibration signals acquired in this paper) was reduced by a factor of three using a down-sampling technique. However, implementing decimation involves aliasing, so a low-pass Chebyshev Type I Finite Impulse Response filter (the filter with the order of 35 and a cut-off frequency of 10 kHz) was used for antialiasing [49]. The output sub-band signal from the lowpass filter (lpf(n), n is denoted as discrete-time) which have frequency spectrums in the range from 0–10 kHz, were then optimized by the ANR-GRS to achieve the optimal sub-band, opt(n), from which twenty-one features were extracted through a feature pool configuration, F(k), for classification by the OAOMCSVM.

### 3.1. Adaptive Noise Reducer–based Gaussian Reference Signal

#### 3.1.1. Adaptive Noise Filtering Technique

##### The Digital Filter

**Figure 6:**
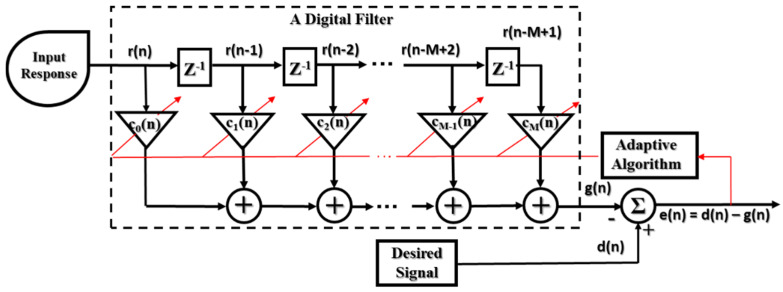
Function block diagram of an adaptive filter.

An adaptive filter combines the operation of a digital filter and an adaptive algorithm. The adaptive algorithm optimizes the coefficient (or weight) of a digital filter by using the feedback signal from the output (error signal) according to the signal condition or performance criteria [38]. Figure 6 illustrates the function of an adaptive filter constructed using a FIR filter and an adaptive algorithm. The output of the FIR filter is calculated as given in Equation (3):

(3) $$g(n)=\sum_{m=1}^{M}c_{m}\left(n\right)r\left(n-m\right)=c^{T}(n)r(n)$$

where, c_m_, m = 0,1, …, M−1 (M is the digital filter length) are the adjustable weights (coefficients) of the filter, which do not depend on the sample time. The weight vector (M × 1) is formed as:

c(n)≡ [c_0_, c_1_, …, c_M−1_]^T^, (4)

and r (n−m), m = 0, 1, …, M−1 are samples of an input signal composed of the vector M × 1:

r(n) ≡ [r(n), r(n−1), …, r(n−M+1)]^T^. (5)

T denotes the transpose operation of the matrix. Then, the error signal e(n) is the difference between the FIR filter response, y(n), and desired signal, d(n), which can be calculated as:

**e**(n) = **d**(n) − **g**(n) = **d**(n) − **c**^T^(n)**r**(n). (6)

A common criterion for tuning the convergence of the weight vector, c(n), is the minimization of the mean-square error (MSE):

  J ≡ E{**e**^2^(n)} = E{[**d**(n)−**c**^T^(n)**r**(n)]^2^} J = **c**^T^(n)R**c**(n) − 2P**c**^T^(n) + E{**d**^2^(n)}, (7)

where, R ≡ E{**r**(n)**r**^T^(n)} is the input autocorrelation matrix, and P ≡ E{**r**(n)**d**(n)} is the cross-correlation vector between the input signal and the desired signal vector.

Equation (7) indicates that the MSE is a quadratic function of the filter weights (c), and its performance surface guarantees that it has a single global minimum MSE corresponding to the optimal vector **c**_o_. The optimal vector **c**_o_ can be found by taking the first derivative of Equation (7) and setting it to zero, the result achieved by Wiener-Hopf equation (assuming that R has an inverse matrix):

**c**_o_ =R^−1^P, (8)

so that the minimum MSE is:

J_min_ = E{**d**^2^(n)} – P^T^**c**_o_. (9)

##### Adaptive Algorithm

The adaptive algorithm is a recursive function to automatically adjust the coefficient vector, c(n), to minimize MSE (J_min_) so that the weight vector converges to the optimum solution, **c**_o_, after iteration loops. Both the LMS and recursive least-squares algorithms can be used to fetch the optimal solution [39], but the LMS is the most broadly used. To calculate the updated weight vector in the recursive loop, the LMS algorithm is based on the steepest-descent procedure using a negative gradient of the instant square error, which was devised by Widrow and Stearns [50] as follows:

c(n+1) = c(n) + µr(n)e(n),(10)

where µ is the step size (or convergence factor) that determines the stability and convergence rate of the LMS algorithm. The algorithm adapts the weight vector to the optimal Wiener-Hopf solution (**c**_o_) given in Eqaution (9) by an iterative process with the convergence factor. The step size is selected in the range [40]:

(11) $$0<µ<\frac{2}{{\mathrm{MS}}_{u}},$$

where $S_{u}$ is the average power of the input signal **r**(n).

##### Adaptive Noise Filtering Technique Applied to a Vibration Signal

**Figure 7:**
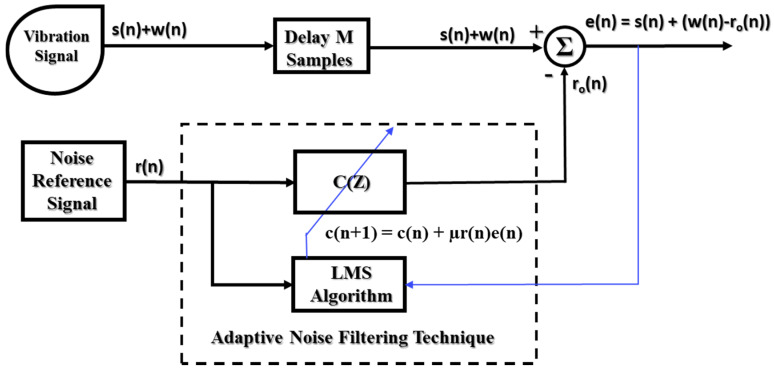
Function block diagram of an adaptive noise filtering technique.

To construct the adaptive noise filter, the noise reference signal and observed signal are applied as the input signal of an adaptive filter (the input response in Figure 6) and the desired signal (d(n) in Figure 6), respectively. The observed signal is the vibration signal acquired from the accelerometer sensor and digitized by the DAQ block reflecting gearbox behavior as expressed by the informative signals (s(n)) and the noise (w(n)), as shown in Figure 7. As explained in Section 1 and Section 2, the informative signals and noise are formed by different sources: the informative signal comes from the vibration of the gear and pinion teeth, whereas the noise comes from the measurement system, unrelated gearbox components, and mechanical resonances. Therefore, the informative signal and noise represent independent processes (E{**s**(n)**w**(n)} = 0). To implement an effective adaptive noise filtering system, the generated noise reference signal, r(n), should meet two conditions (A and B):

(A) The generated noise reference, r(n), and informative signal, s(n), are uncorrelated and independent (E{r(n)s(n)} = 0)
(B) The characteristics of the generated noise reference, r(n), and noise, w(n), are homologous as much as possible.

When those conditions are met, the MSE of the adaptive noise filter can be calculated as follows:

E{**e**^2^(n)} = E{[**s**^2^(n) + (**w**(n) − **r**_o_(n))]^2^}, (12)

where **r**_o_(n) = **c**^T^(n)**r**(n), and the adaptive filter uses an FIR filter,

E{**e**^2^(n)} = E{**s**^2^(n)} + E{[**w**(n) − **c**^T^(n)**r**(n)]^2^}. (13)

The informative signal is independent of both the noise (E{**s**(n)**w**(n)} = 0) and the generated noise reference (E{**r**(n)**s**(n)} = 0). By implementing the LMS adaptive algorithm to adapt the filter coefficient vector, **c**(n), to the optimal vector, **c**_o_, the mean square of the output signal (error signal) approaches the single minimum of the performance surface. From Equation (13), the minimum MSE is taken to be:

(14) $$\underset{c\left(n\right)}{\min}E\left\{e^{2}\left(n\right)\right\}=\;E\left\{s^{2}\left(n\right)\right\}+\underset{c\left(n\right)}{\min}E\left\{{\left[w\left(n\right)-c^{T}\left(n\right)r\left(n\right)\right]}^{2}\right\}$$

Therefore, the output signal of the adaptive noise filtering system carries the complete informative part of the gearbox vibration signal throughout the whole process of algorithm implementation. In addition, the noise integrated into the vibration signal is reduced; in the ideal case, the noise is removed ($\underset{c\left(n\right)}{\min}E\left\{{\left[w\left(n\right)-c^{T}\left(n\right)r\left(n\right)\right]}^{2}\right\}$ = 0). Therefore, for adaptive noise control, we implement the ANR-GRS. 

#### 3.1.2. ANR-GRS 

**Figure 8:**
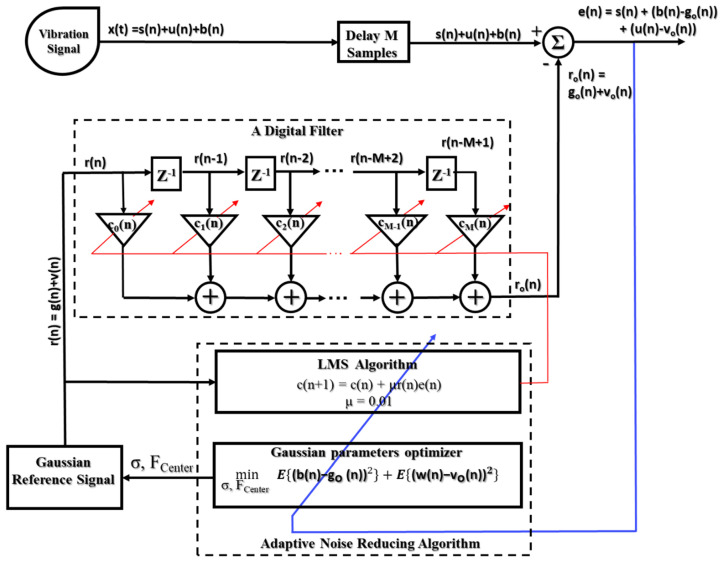
A function block diagram of the ANR-GRS module and parameter adjustments.

**Figure 9:**
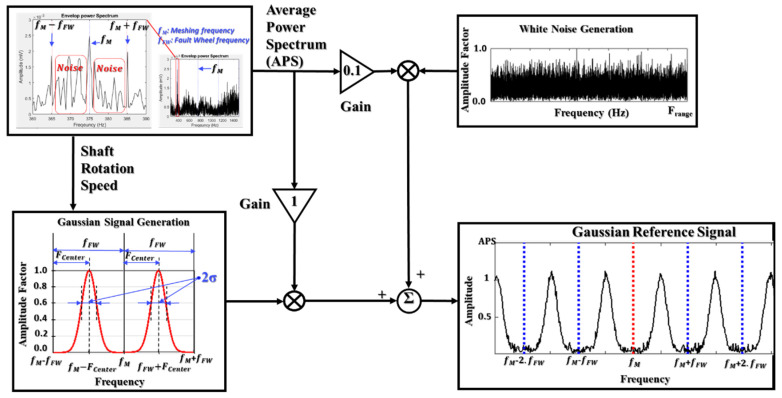
The overall flow chart of GRS signal generation for the ANR-GRS module.

In this paper, the noise (w(n)) in the gearbox vibration signal is divided into two types: white noise (u(n)) and band noise (b(n)). The white noise arises from the measurement system: the amplifier, detector, DC power supply, thermal vibration of the semiconductor atoms, etc. In the frequency domain, the power of the white noise is spread across the whole frequency spectrum of the vibration signal (theoretically, the power of white noise is spread from -∞ to ∞ in the frequency axis) [8]. Band noise, on the other hand, represents noise caused by unrelated components [7]. The frequency harmonics of the band noise are distributed around the informative components of the gear sideband frequency, meshing frequency, and their harmonics. Therefore, the informative signal inside the vibration signal is separately independent of both types of noise. The ANR-GRS module is built using the adaptive noise filtering technique and reference noise-related generation signals, as illustrated in Figure 8. To reduce the white noise, we apply a generated white noise signal with a uniform, random distribution function (v(n)). The oscillation form of the generated white noise is thus analogous to the white noise integrated into the vibration signal. Because its frequency spectrum is within the observed frequency range, the maximum level of the power spectrum average (PSA) of the reference white noise is reduced to less than 10% (10% in this study) of the PSA of the vibration signal to ensure that the informative signal can be eligible for conditions A or B. The GRS (g(n)) is created to adapt to the band noise inside the vibration signal. To make the proposed methodology as an invariant model, the GRS generation module uses the shaft rotation speed (RPM) information from the displacement transducer and the vibration signal as the input parameters. Then, the mean frequency ($F_{\mathrm{Center}}$) and the standard deviation of the GRS are calculated based on the frequency of the defective wheel, which is a function of RPM (the gear frequency in this paper). The GRS window is confined entirely within the frequency space between two consecutive sideband frequencies (a sideband segment), pictorially described in Figure 9, and computed as follows:

(15) $$W_{\mathrm{GRS}}\left(k\right)=\overset{N_{b}}{\underset{k=1}{\sum }}e^{-\frac{{\left(k-F_{\mathrm{Center}}\right)}^{2}}{2\Delta }},$$

where $\Delta \;=\sigma^{2}$ is the variance, σ is the standard deviation of the GRS window, and $F_{\mathrm{Center}}$ is the mean frequency of the GRS window. They function as the frequency of a faulty wheel (the gear frequency, $f_{G}=P.\mathrm{RPM}/G$ in this research).

(16) $$\;F_{Center}=\alpha .f_{FW}.$$

By linearization of the Gaussian function, the standard deviation (the characteristic of a Gaussian distribution) can be approximately calculated as:

(17) $$\sigma =0.318.F_{\mathrm{Center}}=0.318.\alpha .f_{\mathrm{FW}}.$$

$N_{b}$ is the number of frequency bins in a sideband segment and defined as follows:

(18) $$N_{b}=\frac{2N_{s}}{F_{s}}.f_{\mathrm{FW}},$$

where $N_{s}$ is the number of samples of the vibration signal, $F_{s}$ is the sampling frequency of the vibration signal, and $f_{\mathrm{FW}}$ is the frequency of the faulty wheel (the gear frequency, $f_{G}$, in this paper). 

To qualify condition B, the frequency components of a Gaussian window are separated from the informative frequencies (sideband frequencies). A Gaussian window is placed completely inside the space between two continual sideband frequencies in the visualization. Thus, the adaptation process for a band-noise reduction is to preserve the original informative frequency component (significantly reducing the noise components and causing negligible attenuation of the informative components). From Equations (15)–(17) and Figure 9, the coefficient α is selected in the range from 0.25 to 0.75, and the qualified Gaussian window signals are generated using the parameters in the following ranges:

The range of the mean value:

(19) $$0.25.{\;f}_{\mathrm{FW}}\leq {\;F}_{\mathrm{Center}}\leq 0.75.f_{\mathrm{FW}}.$$

The range of the standard deviations of the Gaussian reference signal:

(20) $$\sigma =\{\begin{array}{cc} 0.318.\alpha .f_{FW} & \;\;\;when\;0.25\leq \alpha \leq 0.5\\ 0.318.(1-\alpha ).f_{FW} & \;\;\;\;when\;0.5<\alpha \leq 0.75 \end{array}$$

Therefore, the implementation of a stepping adjustment in the coefficient α drives a change in the mean value and standard deviation (the position and shape) of the Gaussian window, which defines the condition for fetching the optimal Gaussian window, as illustrated in Figure 9.

#### 3.1.3. The Process for Calculating the Optimized Subband

First, the ANR-GRS algorithm germinates the initial parameters for the Gaussian signal generation module: starting value of α=0.25 in this paper, adaptive filter (M-tap, M = 40 in this study), coefficient vector **c**(n) = [0, 0, …, 0], and step size µ (µ = 0.01). The parameter α is scanned in the range [0.25 0.75] in steps of 0.01 in company with the input rotation speed (RPM) to compute the $F_{\mathrm{Center}}$ (mean value) and standard deviation (σ) using Equations (16) and (17). To generate the specific GRS needed for the reference input of the adaptive filter **r**(n), the output of the adaptive filter is connected to the minus port of the summation module, **r**_o_(n). The vibration signal, which contains both the informative component and noise, is entered as the desired input and delayed for M sampling time steps to be compatible with the delayed processing of the FIR digital filter. The LMS algorithm adjusts the coefficient vector to receive the LMS of the error, which is the output of the summation module. The output error signal, which has LMS (and to which the optimal coefficient vector is set), is pushed into the set of proposed optimized sub-bands.

**Figure 10:**
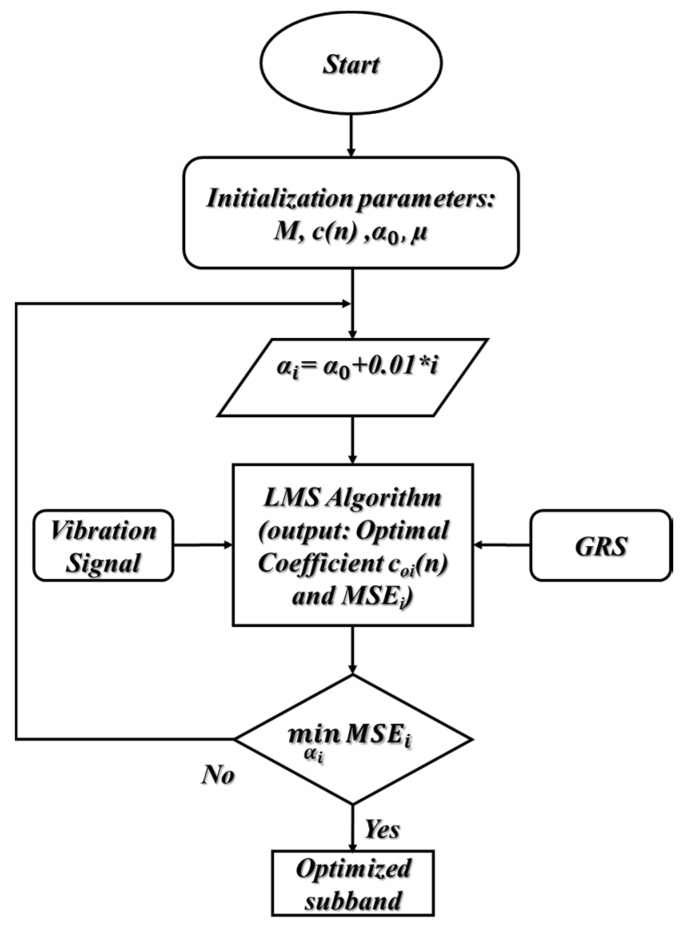
The algorithm flow chart of the ANR-GRS module.

Finally, the algorithm calculates the mean square value of each sub-band in that set and then selects the sub-band with the minimum value as the optimized sub-band and output of the AND-GRS module (Figure 10).

### 3.2. Feature Pool Configuration 

We found the ANR-GRS methodology to be highly effective in reducing most of the noise components from a 1-s raw vibration signal while leaving the information about gearbox faults intact. The optimized sub-band output from the ANR-GRS, i.e., the “clean” signal presenting the characteristics of the gearbox component vibration with trivial noise effects, carries the intrinsic fault symptoms of the cut tooth defects. We then use those optimal sub-bands, rather than the raw 1-s vibration signals, to extract features. According to Caesarendra et al. [51], the statistical parameters from the time and frequency domains of the signal are congruent and subservient for fault classification using machine learning. Table 3 displays twenty-one features, eighteen time-domain features (e.g., root means square, square mean root, kurtosis, skewness, margin, impulse, and peak-to-peak value) and three frequency-domain features (root mean square frequency, frequency center, and root variance frequency) for each optimal sub-band. The feature pool dimensionality is N_HS_ × N_1-SEC_ × N_F_, where N_HS_ is the number of gearbox health states (number of classes) that need to be classified (4 classes in this study: healthy, pinion tooth cut 10%, pinion tooth cut 30%, and pinion tooth cut 50%), N_1-SEC_ is the number of 1-s samples of each class (300 in this study), and N_F_ defines the number of features (21 in this study). Therefore, groups of 21-feature vectors were considered as the validating input dataset for our proposed intelligent fault-detection method based on a multiclass SVM.

### 3.3. Gearbox Fault Classification Using a Multiclass SVM Classifier

The principle operation of an SVM is based on the statistical learning theory of Vapnik [45] and quadratic programming [46]. It was actually designed to classify binary datasets by finding the optimal plane, generally called the *hyperplane*, with the largest margin-gap separating it from both binary classes. Let {($x_{m}$, $y_{m}$), m=1, 2, …, M} be the given training dataset with M samples, where each sample data $x_{m}\in {\mathbb{R}}^{D}$, ${\mathbb{R}}^{D}$ is a D-dimensional feature vector, and $y_{m}$ ($y_{m}\in \left\{-1,+1\right\}$) are the class labels. The SVM is used to find a set of linearly separable hyperplanes between two classes and maintain the maximum distance (called the *margin*) from both of them.

The hyperplane, denoted as w, is determined as the maximized width of the margin and the minimized structural risk, given by:

(21) $$\left(w,b\right)=\underset{w,b}{\mathrm{argmin}}\frac{1}{2}w^{T}w+C\overset{M}{\underset{m=1}{\sum }}\xi_{m},$$

subject to: $y_{m}(w^{T}\psi \left(x_{m}\right)+b)\geq \;1-{\;\xi }_{m}$, ∀m = 1, 2, …, M; $-{\;\xi }_{m}\leq \;0$, ∀m = 1, 2, …, M 

**Figure 11:**
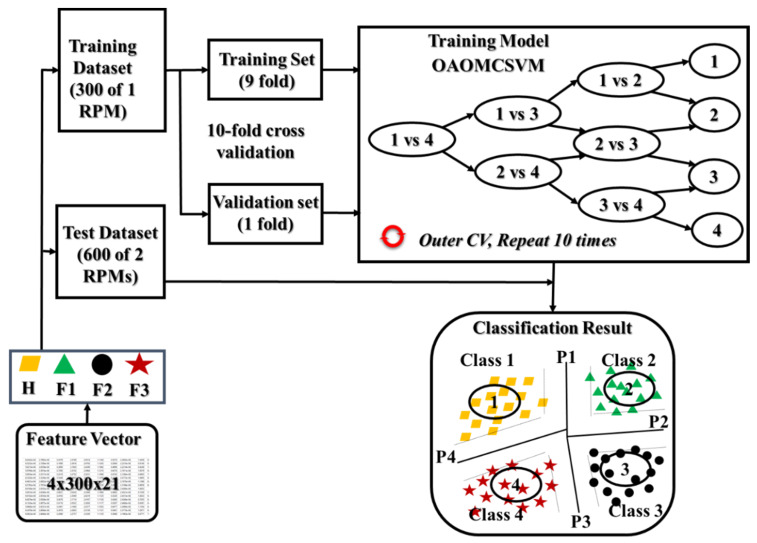
The classification methodology of OAOMCSVM.

Here, b is bias, C is the trade-off parameter, $\xi \;=\left\{\xi_{1},\xi_{2},\ldots ,{\;\xi }_{N}\right\}$ is the set of slack variables, and ψ(.) is a feature vector in the expanded feature space. Equation (21) can be solved by applying the Lagrange duality solution [44] as shown below:

(22) $$\underset{\alpha }{\mathrm{argmax}}w\left(\alpha \right)=\overset{M}{\underset{m=1}{\sum }}\alpha_{m}-\frac{1}{2}\overset{M}{\underset{m=1}{\sum }}\overset{M}{\underset{k=1}{\sum }}\alpha_{m}\alpha_{k}y_{m}y_{k}\psi^{T}\left(x_{m}\right)\psi \left(x_{k}\right),\;$$

subject to: $\sum_{m=1}^{M}\alpha_{m}y_{m}=0$, $0\;\leq b_{m}\leq \;C$, ∀m = 1, 2, …, M where, $\alpha_{m}$ and $\alpha_{k}$ are Lagrange multipliers, $x_{m}$ and $x_{k}$ are two input training vectors, and $K(x_{m},x_{k}$) = $\psi^{T}\left(x_{m}\right)\psi \left(x_{k}\right)$ is a kernel function used to map the input data space into a higher-dimensional feature space. Several kernel functions, such as linear, polynomial, Gaussian, radial basis, and sigmoid functions, can be used in SVM classification methods. Countless classification applications have more than two classes in their datasets and thus require a solution beyond the binary SVM just described. Multiclass SVMs have been developed to classify datasets of N different classes (N > 2), and they use one of three structures: one-against-one, one-against-all, and hierarchical. Among those structures, OAOMCSVM requires more classifiers than the others, but it also has the most reliable classification accuracy [45]. Therefore, we use OAOMCSVM, illustrated in Figure 11, in the methodology proposed in this paper.

## 4. Experimental Results

To verify the advantage of the ANR-GRS module in the proposed methodology, we implemented experiments in two technological zones, signal processing and features dataset classification, and compared our results with those from conventional methodologies.

### 4.1. Signal Processing Experimental Results

The 1-s vibration signals acquired using the experimental testbed described above contained the phase and amplitude modulation signals bearing information about the health states of a gearbox. To investigate the effectiveness of the proposed noise reduction technique, the experimental dataset was collected under various shaft rotation speeds that are equal to 300, 600, 900, and 1200 RPM, respectively. The vibration signals output from the accelerometer are analog, so they were digitized with a high holding sample frequency of 65536 Hz to gather as much information (and noise) as possible on the wideband PCI-based data acquisition board (Table 1). Each 1-s digital vibration signal was down-sampled three times, incorporating a lowpass filter for antialiasing to output a digital vibration signal realistically compatible with the working range frequency of the accelerometer (0–10 kHz, Table 1). Then, the vibration signal was input into the ANR-GRS module (Figure 5). The shaft rotation speed (RPM), measured by the displacement transducer, was observed by the ANR-GRS module according to appropriate vibration signal data to generate the Gaussian reference signal. The optimal subbands were the output of the ANR-GRS module.

**Figure 12:**
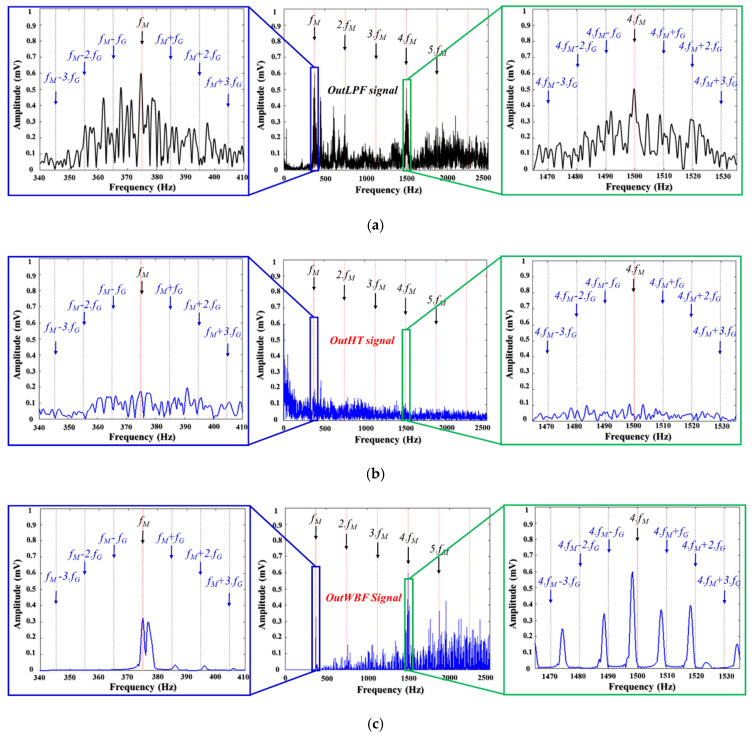

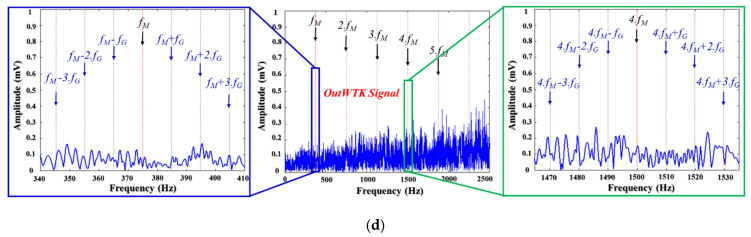
The frequency spectrum analysis for the state-of-the-art methodologies: (**a**) the input signal, (**b**) the output signal from the Hilbert transform module, (**c**) the output signal from the window bandpass filter module, and (**d**) the output signal from the wavelet transform WTK module.

**Figure 13:**
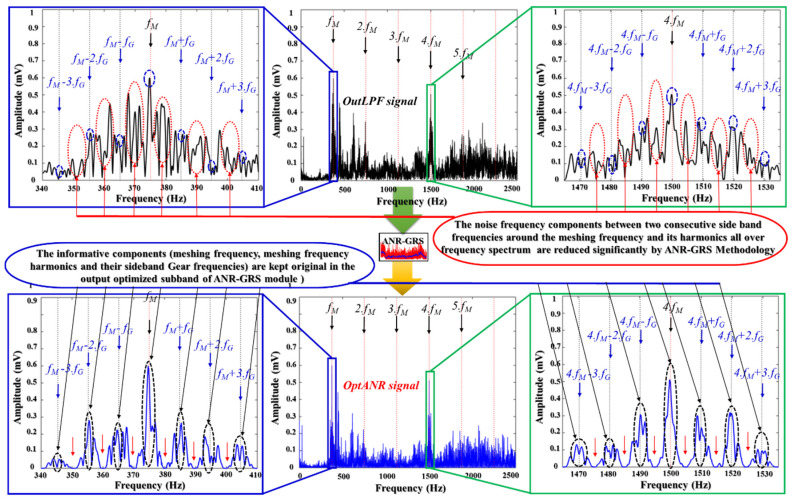
Frequency spectrum analysis of the input and output signal of the ANR-GRS module.

To demonstrate the superiority of the ANR-GRS technique, we compared its optimized subbands with the outputs of other signal processing approaches for noise reduction: the Hilbert transform (HT), window bandpass filter (WBF), and wavelet transform with optimal subband-based maximum kurtosis (WTK). We tested those approaches by replacing the ANR-GRS module with them. Figure 12 illustrates the frequency spectra compared with the input vibration signal. Figure 12a shows the output of a lowpass filter that received a 1-s vibration sample with 900 RPM (15 Hz) of fault type 2 (meshing frequency, $f_{M}=P.\mathrm{RPM}=25.15=375\;\mathrm{Hz}$ and sideband gear frequency, $f_{G}=P.\mathrm{RPM}/G=9.87\;\mathrm{Hz}$, shown as lpf(n) in Figure 5 and labeled as the OutLPF signal in Figure 12a). The output signals from the noise-reduction modules are shown in Figure 12b (OutHT signal), Figure 12c (OutWBF signal), Figure 12d (OutWTK signal), and Figure 13, the proposed ANR-GRS (OptANR signal). The three conventional methods (HT, WBF, WTK) changed the outLPF signal into different shapes and types (the outLPF signal is an amplitude and phase modulation signal) regardless of the fault information (meshing frequency and its harmonics and sideband gear frequencies). HT exalted the area of the low-frequency components, whereas WTK fortified the high-frequency components in the frequency spectrum (Figure 12b,d). WBF was better than the HT and WTK methods because it filtered the noise in some of the meshing frequency harmonics and sideband gear frequencies, but it also reduced or removed significantly informative frequency components (Figure 12c). The outANR signal (Figure 13), the output signal from the ANR-GRS module proposed here, fulfilled the needs of signal processing: reducing the noise components and preserving the original informative components. It made the vibration signal from the gearbox “cleaner” (lowered the noise) and approached the characteristics of the gearbox vibrations signal presented in Section 2.1. This comparison verifies that our accurate is a suitable technique for reducing the noise in gearbox vibration signals and returning an honest reflection of the health states of a gearbox along an electronic signal path.

### 4.2. Classification Results

The classification performance of the proposed methodology is evaluated in two experiments. At the beginning, the feature sets of the dimensions 4 × 300 × 21 (300 samples from each of four health states, as shown in Table 2) for each speed (in this experiment, 300 RPM, 600 RPM, 900 RPM, and 1200 RPM) were selected. Then, in the first experiment, the dataset is created by merging the data of four available health states under different rotating speeds resulting in the new feature set of the dimensionality 4800 × 21. This dataset was then randomly divided into a train and a test sets at ratio 8:2 for training and testing OAOMCSVM classifier and general evaluation of the proposed fault diagnosis methodology. 

Furthermore, to prove the robustness of the proposed ANR-GRS technique, the second experiment is performed where the classifier is trained on data (i.e., feature sets) corresponding to single rotating speed and tested by data instances collected under other speeds. Specifically, feature set for a single rotating speed was used as a training set (for instance, feature set corresponding to 300 RPM with the dimensionality of 4 × 300 × 21), and the feature sets corresponding to two other speeds were used for testing (for instance, feature set corresponding to two other speeds 600 RPM and 900 RPM with the dimensionality of 4 × 600 × 21).

Those processes were run four times. To construct the training model for classification, k-fold cross-validation (k-cv) was used to estimate the accuracy of the generalized classification [52]. In k-cv, the set of samples in the feature vector is split randomly into k mutual folds (k=10 in this study), denoted as C1, C2, …, Ck. The classification OAOMCSVM operates on k-times of the accuracy estimation.

Some folds {Cj} (a random subset from k folds) are used as a training set, and the rest are used as a validation set and alternative iteration k times. More specifically, for each speed, 300 feature vectors for each health state in the training set were partitioned into ten folds (each fold containing 30 randomly chosen feature vectors (30 × 21) for each health state); 9 of those folds were used for training, and the 1 remaining fold was used for validation. That process was repeated 10 times until all folds had been used as the validation set. The final measure of performance in the training model is the average value of the accuracies attained in each fold. These data are then used as the testing dataset (which was not used at all in the training process) to verify the OAOMCSVM method and provide the final classification result.

**Figure 14:**
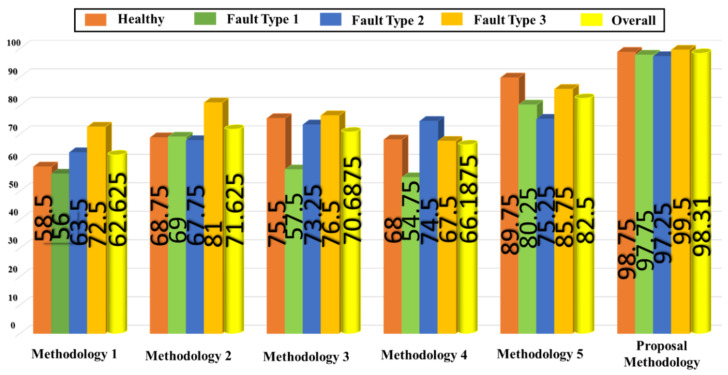
The accuracy of each class and the average accuracy of the state-of-the-art methodologies and the proposed ANR-GRS methodology.

We also used the OAOMCSVM classification method to classify the feature pool configuration datasets extracted from the comparison signal processing methodologies: the raw vibration signal (lowpass filter output signal) extraction (methodology I), HT, (methodology II), WBF (methodology III), WTK (methodology IV), and the IMFs and residuals from the EMD (methodology V). The implementation of those methodologies for achieving the classification result for the four health states complied strictly with the conditions used with input from the ANR-GRS module just described. To estimate the classification result between methodologies (the proposed method and others), all twenty-one features of the vibration signal were used as input feature vectors for the OAOMCSVM module to ensure that the most informative features for and from each methodology were used fairly for the classification. The classification results of the state-of-the-art methodologies and the proposed ANR-GRS methodology obtained during two experiments are shown in Table 4 and Table 5 and Figure 14 to visualize the results tabulated in Table 5. Those classification accuracies were computed as follows:

(23) $$C_{\mathrm{accuracy}}=\frac{\sum_{L}N_{\mathrm{TP}}}{N_{\mathrm{samples}}}.100\%$$

where *L* is the number of categories (*L* = 4 as four health states), $N_{\mathrm{TP}}$ is the number of true positives (the number of fault samples in category *i* that are correctly classified as class *i*), and $N_{\mathrm{samples}}$ is the total number of samples used to estimate the performance of the proposed methodology.

Table 4 illustrates that the proposed technique significantly outperforms its counterparts when it is trained on the data instances corresponding to all available speeds and achieving the highest accuracy of 99.7%.

Table 5 demonstrates that the proposed approach using ANR-GRS also yielded the highest average classification accuracies (98.31%) in comparison with the other five state-of-the-art signal processing methodologies when it is trained and validated on datasets corresponding to separate rotating speeds. 

The methodology I extracted the feature vectors of all four speeds for classification by the OAMCSVM directly from the raw vibration signal (OutLPF signal), in which non-linear and non-stationary signals drown out the informative signal. Accordingly, those results are distributed chaotically among the four classes, producing the lowest accuracy among the 6 methodologies (62.63%). For methodologies II, III, and IV, the vibration signals change with the different characteristics of the gearbox vibration signal (its amplitude and phase modulation signal), so their classification accuracy is also low, around 70%. Methodology V (the EMD technique) is outstanding in comparison with the first four approaches (82.5%) because it extracts IMFs, which contain fault-related information to better discriminate between classes. However, IMFs can be mistakenly extracted from noise components, which damaged the accuracy compared with the ANR-GRS technique by around 15%. 

**Figure 15:**
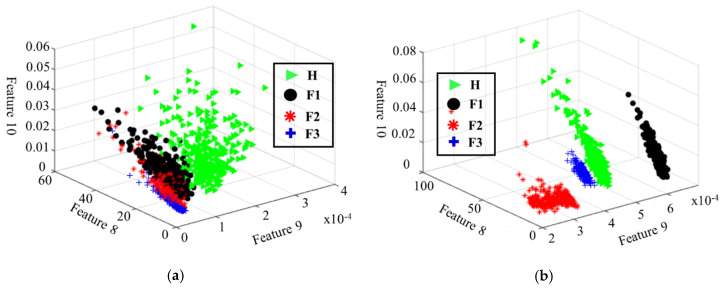
Three-dimensional visualization of features extracted from (**a**) the input signal of the ANR-GRS module and (**b**) the output signal of the ANR-GRS module.

In addition, as a quantitative evaluation, we present the space distribution in a 3-dimensional visualization (Figure 15) of samples belonging to four classes based on some features extracted from the outLPF signal and the outANRsignal (signals before and after using the ANR-GRS technique, respectively). The features of the outANR signal show better separation and clustering for different health states of the gearbox fault diagnosis experimental scheme. Samples from the same class are more closely clustered, whereas samples from different classes are discriminated and easy to classify. On the contrary, before using the ANR-GRS, the features of different classes overlap, making it difficult to distinguish the fault classes.

**Figure 16:**
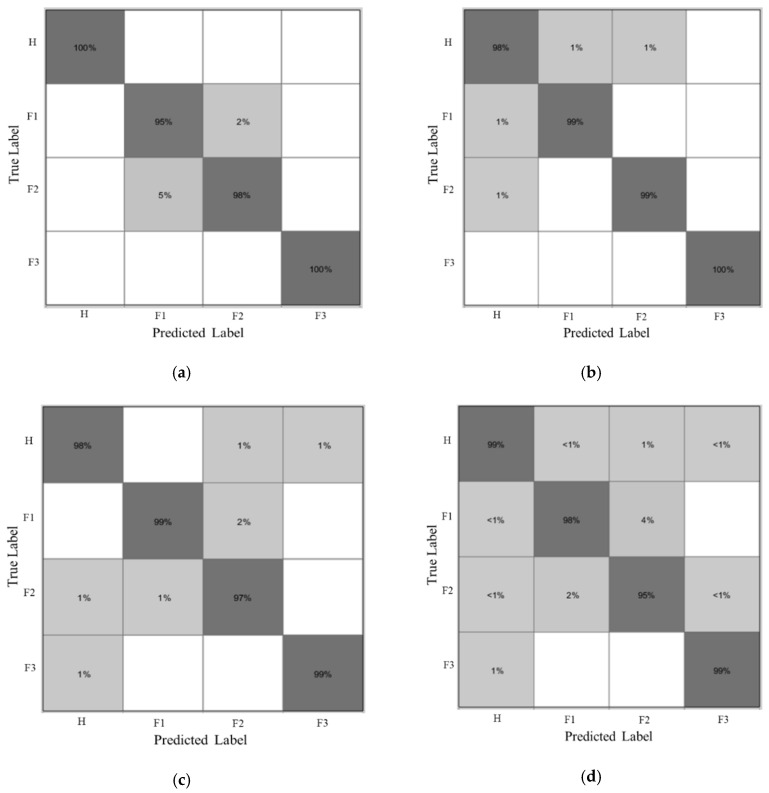
Confusion matrixes for four classification cases according to the speed dataset input for training: (**a**) 300 RPM, (**b**) 600 RPM, (**c**) 900 RPM, (**d**) 1200 RPM.

Moreover, confusion matrixes are shown in Figure 16 to demonstrate the reliability of the varying-speed gearbox fault diagnosis methodology using the ANR-GRS module for effective noise reduction. Using real-time tracking of the rotation speed (RPM) of a gearbox system, the ANR-GRS generated speed-related function signals for real-time tracking of speed-dependent noise components, and the optimized output signal was unaffected by speed during classification. 

## 5. Conclusions

In this study we propose a reliable fault diagnosis methodology for gearbox systems under varying speed conditions. It integrates adaptive noise control to significantly reduce noise with machine learning classification to classify the fault states of the gearboxes. First, we created a set of Gaussian reference signals that are a function of the rotation speed and consist of many noise components such as white noise and band noise that are correlated to the parasitic noise in the vibration signals and independent of the intrinsic informative components. Then, we applied those GRSs to an adaptive noise control technique that produced an optimal sub-band as output for each 1-s vibration sample. The most optimal sub-bands were then used in the feature pool configuration to extract feature vectors, and an OAOMCSVM was used for classification. The experimental results indicated that the proposed gearbox fault diagnosis methodology achieved the highest classification accuracies in both experiments that are equal to 99.7% and 98.31% while significantly outperforming the counterpart state-of-the-art methodologies used for the comparison. In future research, we will continue improving the robustness of the proposed methodology and investigate it’s applicability to the real-time fault diagnosis scenarios. 

## Figures and Tables

**Table 1:** Specification of the sensors and data acquisition system.

Device	Specification
**Vibration sensor (Accelerometer 622B01)**	Sensitivity (V/g): 10.2 mV/(m/s^2^)
	Operational frequency range: 0.42 to 10 kHz
	Resonant frequency: 30 kHz
	Measurement range: ± 490 m/s^2^
**4- Channel DAQ PCI Board**	18-bit 40MHz AD conversion, a sampling frequency of 65.536 kHz is used for each of two channels simultaneously
**Displacement transducer**	Distance from the head of a transducer to a hole: 1.0 mm
	Diameter of a hole: 12.80 mm
	Sensitivity: 0 to −3dB
	Frequency response: 0–10 kHz

**Table 2:** A detailed description of the fault types and dataset.

Gearbox Health State	Description	Number of 1-s Data Samples Acquired for each Rotation Speed				Sampling Frequency (Hz)
		300 RPM	600 RPM	900 RPM	1200 RPM	
**Healthy** **(H)**	No seeded fault in the teeth of a gearbox	300	300	300	300	65536
**Fault type 1 (F1)**	Pinion tooth cut 10% (0.9 mm)	300	300	300	300	65536
**Fault type 2 (F2)**	Pinion tooth cut 30% (2.7 mm)	300	300	300	300	65536
**Fault type 3 (F3)**	Pinion tooth cut 50% (4.5 mm)	300	300	300	300	65536

**Table 3:** Definition of statistical features in the time and frequency domains.

Features	Equations	Features	Equations	Features	Equations
Peak	Max(|s|)	Shape factor	$\frac{s_{rms}}{\frac{1}{N}\sum_{n=1}^{N}\left|s_{n}\right|}$	Mean ($\bar{s}$)	$\frac{1}{N}\overset{N}{\underset{n=1}{\sum }}s_{n}$
Root mean square (s_rms_)	$\sqrt{\frac{1}{N}\overset{N}{\underset{n=1}{\sum }}s_{n}^{2}}$	Entropy	$-\overset{N}{\underset{n=1}{\sum }}p_{n}.log_{2}\left(p_{n}\right)$	Shape factor square mean root	$\frac{s_{srm}}{\frac{1}{N}\sum_{n=1}^{N}\left|s_{n}\right|}$
Kurtosis	$\frac{1}{N}\overset{N}{\underset{n=1}{\sum }}\left(\frac{s_{n}-\bar{s}}{\sigma }\right)$	Skewness	$\frac{1}{N}\overset{N}{\underset{n=1}{\sum }}{\left(\frac{s_{n}-\bar{s}}{\sigma }\right)}^{3}$	Margin factor	$\frac{\max\left(s\right)}{s_{smr}}$
Crest factor	$\frac{\mathrm{Max}\left(\left|s\right|\right)}{s_{rms}}$	Square mean root (s_smr_)	${\left(\frac{1}{N}\overset{N}{\underset{n=1}{\sum }}\sqrt{\left|s_{n}\right|}\right)}^{2}$	Peak to peak	max(s)−min(s)
Clearance factor	$\frac{\mathrm{Max}\left(\left|s\right|\right)}{s_{smr}}$	5th normalized moment	$\frac{1}{N}\overset{N}{\underset{n=1}{\sum }}{\left(\frac{s_{n}-\bar{s}}{\sigma }\right)}^{5}$	Kurtosis factor	$\frac{Kurtoris}{s_{rms}^{4}}$.
Impulse factor	$\frac{\mathrm{Max}\left(\left|s\right|\right)}{\frac{1}{N}\sum_{n=1}^{N}\left|s_{n}\right|}$	6th normalized moment	$\frac{1}{N}\overset{N}{\underset{n=1}{\sum }}{\left(\frac{s_{n}-\bar{s}}{\sigma }\right)}^{6}$	Energy of signal	$\overset{N}{\underset{n=1}{\sum }}s_{n}^{2}$
Frequency center (FC)	$\frac{1}{N_{f}}\overset{N_{f}}{\underset{f}{\sum }}S\left(f\right)$	Root mean square frequency	$\sqrt{\frac{1}{N_{f}}\overset{N_{f}}{\underset{f}{\sum }}S{\left(f\right)}^{2}}$	Root variance frequency	$\sqrt{\frac{1}{N_{f}}\overset{N_{f}}{\underset{f}{\sum }}{\left(S\left(f\right)-FC\right)}^{2}}$

Here is an input signal (i.e., optimized subband), N is the total number of samples, S(f) is the magnitude response of the fast Fourier transform of the input signal s, $N_{f}$ is the total number of frequency bins, $\sigma =\sqrt{\frac{1}{N}\sum_{n=1}^{N}{\left(s_{n}-\bar{s}\right)}^{2}}$, and $p_{n}=\frac{s_{n}^{2}}{\sum_{n=1}^{N}s_{n}^{2}}$.

**Table 4:** Classification results for state-of-the-art methodologies and the proposed ANR-GRS methodology by a combination dataset of different speeds.

Methodology	OAOMCSVM (4800 Samples)		Accuracy (%)				
	Training Set (80%)	Test Set (20%)	Healthy	Fault Type 1	Fault Type 2	Fault Type 3	Overall (%)
**I**	3840	960	59	73	69	75	69.0
**II**	3840	960	84	80	67	83	78.30
**III**	3840	960	92	89	76	83	84.6
**IV**	3840	960	85	87	58	74	73.10
**V**	3840	960	92	89	88	94	90.80
**ANR-GRS**	3840	960	100	99	99	100	99.70

**Table 5:** Classification results for state-of-the-art methodologies and the proposed ANR-GRS methodology by observation of separated speed dataset.

Methodology	OAOMCSVM (10-Fold Cross Validation)		Accuracy (%)				
	Training Set (300 Samples)	Test Set (600 Samples)	Healthy	Fault Type 1	Fault Type 2	Fault Type 3	Overall (%)
I	300 RPM	600 RPM, 900 RPM	53	78	69	52	63
	600 RPM	900 RPM, 1200 RPM	74	47	53	80	63.5
	900 RPM	600 RPM, 1200 RPM	54	46	64	81	61.25
	1200 RPM	300 RPM, 600 RPM	53	53	68	77	62.75
	Overall by health states		58.5	56	63.5	72.5	**62.63**
II	300 RPM	600 RPM, 900 RPM	51	99	63	85	74.5
	600 RPM	900 RPM, 1200 RPM	75	67	64	72	69.5
	900 RPM	600 RPM, 1200 RPM	75	48	70	83	69
	1200 RPM	300 RPM, 600 RPM	74	62	74	84	73.5
	Overall by health states		68.75	69	67.75	81	**71.63**
III	300 RPM	600 RPM, 900 RPM	75	58	69	93	73.75
	600 RPM	900 RPM, 1200 RPM	74	70	80	84	77
	900 RPM	600 RPM, 1200 RPM	70	49	72	63	63.5
	1200 RPM	300 RPM, 600 RPM	83	53	72	66	68.5
	Overall by health states		75.5	57.5	73.25	76.5	**70.69**
IV	300 RPM	600 RPM, 900 RPM	64	74	87	63	72
	600 RPM	900 RPM, 1200 RPM	82	49	72	64	66.75
	900 RPM	600 RPM, 1200 RPM	63	47	69	76	63.75
	1200 RPM	300 RPM, 600 RPM	63	49	70	67	62.25
	Overall by health states		68	54.75	74.5	67.5	**66.19**
V	300 RPM	600 RPM, 900 RPM	77	94	72	89	83
	600 RPM	900 RPM, 1200 RPM	90	82	91	82	86.25
	900 RPM	600 RPM, 1200 RPM	94	80	69	85	82
	1200 RPM	300 RPM, 600 RPM	98	65	69	83	78.75
	Overall by health states		89.75	80.25	75.25	84.75	**82.5**
ANR-GRS	300 RPM	600 RPM, 900 RPM	100	95	98	100	98.25
	600 RPM	900 RPM, 1200 RPM	98	99	99	100	99
	900 RPM	600 RPM, 1200 RPM	98	99	97	99	98.25
	1200 RPM	300 RPM, 600 RPM	99	98	95	99	97.75
	Overall by health states		98.75	97.75	97.25	99.5	**98.31**
